# Crack resistance of a noble green hydrophobic antimicrobial sealing coating film against environmental corrosion applied on the steel–cement interface for power insulators†

**DOI:** 10.1039/d2ra00747a

**Published:** 2022-03-31

**Authors:** Simpy Sanyal, Ramachandran Chelliah, Taeyong Kim, Matheus Rabelo, Deog-Hawn Oh, Duy Phong Pham, Junsin Yi

**Affiliations:** Department of Electrical and Computer Engineering, Sungkyunkwan University Suwon 16419 Republic of Korea pdphong@skku.edu; Interdisciplinary Program in Photovoltaic System Engineering, Sungkyunkwan University Suwon 16419 Republic of Korea; College of Information and Communication Engineering, Sungkyunkwan University Suwon 16419 Republic of Korea junsin@skku.edu; Department of Food Science and Biotechnology, College of Agriculture and Life Sciences, Kangwon National University Chuncheon 24341 Republic of Korea; Kangwon Institute of Inclusive Technology (KIIT), Kangwon National University Chuncheon 24341 Republic of Korea

## Abstract

Due to their great load-bearing capabilities, steel–cement interface structures are commonly employed in construction projects, and power utilities including electric insulators. The service life of the steel–cement interface is always decreasing owing to fracture propagation in the cement helped by steel corrosion. In this paper, a noble crack-resistant solution for steel–cement interfaces utilized in hostile outdoor environments is proposed. A Ce-rich, homogeneous, and thick hydrophobic sealing coating (HSC) is developed on the steel–cement interface after 60 minutes of immersion in a 60 000 ppm CeCl_3_·7H_2_O sealing coating solution. The specimens treated with optimized HSC film demonstrate fissure filling, lowest corrosion current (*I*_corr_) 2.3 × 10^−7^ A cm^−2^, maximum hardness (109 Hv), oxide-jacking resistance (40 years), hydrophobic characteristics, carbonation resistance, and bacterial corrosion resistance, resulting in a crack-free steel–cement interface. This work will pave the way for a new branch of environmentally acceptable coatings for the construction and power industries.

## Abstract 2

Due to their great load-bearing capabilities, steel–cement interface structures are commonly employed in construction projects, and power utilities including electric insulators.

## Graphical abstract

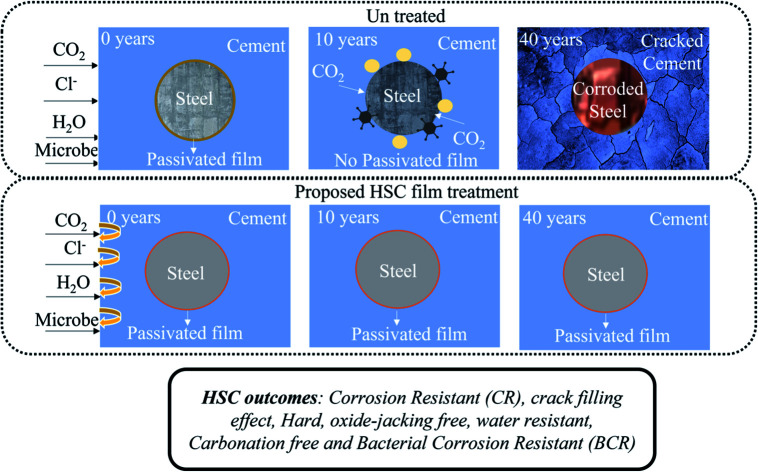

## 1. Introduction

Insulators are essential components of electric power transmission networks. High-voltage insulators offer mechanical support and electrical isolation to wires from the ground structure. The cement acts as a binder for the shell and metal fittings owing to its good mechanical properties. The insulator pin and cement carry tensile stress for insulators. However, corrosion is one of the principal causes of service life and durability issues in high-voltage insulators. The insulator hardware (pin) naturally interacts with oxygen in a very alkaline (pH 13) environment formed by cement hydration surrounding it, forming a thin oxide passive layer on its surface that functions as a physical barrier against corrosion.^1–3^ Admittedly, in harsh environments such as airborne sea spray, sea storms, acid rain, and corrosion-inducing microbes, the passivating film formed on the insulator steel (pin) that protects the steel (pin) from corrosion may be lost due to Cl^−^ ion diffusion and pH, resulting in accelerated corrosion. Furthermore, CO_2_ and H_2_O can penetrate the cement *via* pores and react with Ca(OH)_2_ to produce CaCO_3_ and H_2_O_2_, resulting in a pH reduction in the concrete pore solution. The passive film may dissolve readily into the concrete pore solution, exaggerating steel (pin) corrosion.^4^

A variety of microorganisms, including bacteria, have been discovered to contribute to corrosion by producing a biofilm. A group of researchers recently revealed that *Acidithiobacillus ferroxidans* (ATCC 23270), and *Thiobacillus organoparus* Markosian (27977) caused significant corrosion (bioleaching) to high-grade stainless steel. Acid producing bacteria (APB) have long been recognized to induce corrosion by producing organic acid, which lowers the pH beneath their biofilms. The pH beneath the biofilm might be significantly lower (by two units or more) than the pH in the bulk fluid. When combined with metal oxidation and a suitably low pH, proton attack is thermodynamically efficient. Furthermore, planktonic cells can contribute to corrosion by releasing protons that aid in the maintenance of an acidic environment and accelerate the rate of corrosion.^5–7^

**Fig. 1:**
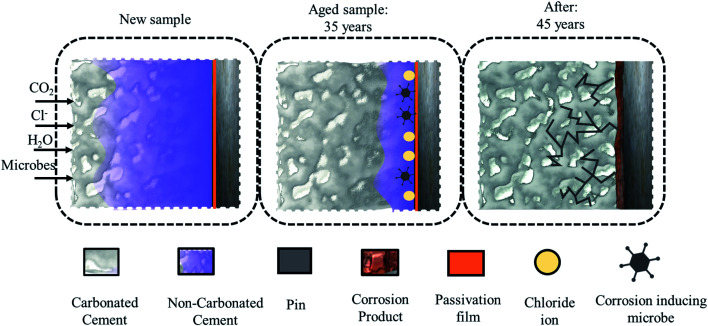
The sequence of propagation of a crack in cement due to carbonation, chloride, and corrosion-inducing microbes.

The corrosion byproducts formed due to corrosion of the steel (pin) apply bursting pressure, which causes radial cracks in the cement (see Fig. 1) and finally the porcelain shell. The corrosion of the insulator steel (pin) may also reduce the cross-sectional or mechanical strength of the steel (pin)–cement interface, leading to sudden ejection of the pin. In addition, once the cement has been substantially moistened to create an electrolytic route, Cl^−^ absorbed in the pore solution can infiltrate *via* the porous network and intrinsic microcracks, finally favoring crack propagation through the cement and porcelain (shell) surface.^8–11^ Thus, preventative measures are required to preserve the steel (pin)–cement interface and the structural integrity of the insulator.

Numerous methods have been discussed in research articles for protecting steel–cement interfaces against cracking. These include cathodic protection, corrosion inhibitors, and anti-corrosion coatings on the metal surface.^8–12^ All previous approaches based on Ce or other materials were focused only on the metal parts and studied the influence on cement/concrete. Nonetheless, the majority of commercial coatings available for the construction/power industries in the market are non-biodegradable and carcinogenic, unlike the proposed treatment. In addition, some previous studies focused on approaches to enhance the durability of concrete alone.^11,12^ There have been no reports of using a cerium-based hydrophobic coating on both steel and cement to increase the endurance of steel–cement contact.

Cerium-based oxides have the potential to be environmentally acceptable materials for use in technical applications such as corrosion prevention, resistance to bacterial-induced corrosion, super-hydrophobic coatings, and thermal barrier coatings.^10–14^ The cerium chemical conversion coatings are the most typically employed to manufacture corrosion protection coatings on metal substrates. They are promising candidates to replace polymer or chromium-based coatings. The stated mechanism suggests that the conversion coatings provide active corrosion protection by interacting with the substrate, which diffuses in the presence of a contaminated environment (low pH), leading to the formation of an amended layer that actively inhibits corrosion *via* Ce(iii)/Ce(iv) redox reactions all along the coating/substrate interface.^13–16^

This current study focuses on the development of a new hydrophobic Ce-based HSC coating for the steel (pin)–cement interface of an insulator to protect against chloride, bacterial-induced corrosion, and carbonation of the steel (pin)–cement interface, resulting in improved crack resistance of the pin–cement–porcelain interface. This sort of approach has not been discussed previously for power/construction industries. The major functional properties (anti-corrosion, fissure filling effect, water, carbonation resistance and anti-bacterial effect) of the developed HSC film were sequentially analyzed based on structural characterization (scanning electron microscopy (SEM), secondary ion spectroscopy (SIMS)); material characterization (X-ray photoelectron spectroscopy (XPS)); functional anticorrosion efficacy (potentiodynamic polarization curves (PDC)), mechanical strength (Vickers hardness test), anti-oxide jacking efficacy (2D simulation), contact angle measurements (hydrophobic characteristics), universal indicator tests (carbonation resistance of the HSC film) and antimicrobial with anti-adhesion efficacy of the HSC coating based on an in-use test, well diffusion method, and confocal three-dimensional scanning image analysis.

## 2. Experimental

### 2.1 Materials

The Korea Electric Power Corporation (KEPCO) supplied the insulator specimens with a (galvanized steel) pin length of 10.7 cm and a zinc coating of 180 μm. The pins and cement (PC 42.1) surrounding the pin were cut into 2 × 2 × 1 cm^2^ cubic sheets using a waterjet. All the chemicals such as isopropyl alcohol (IPA), Turco 6849, hydrogen peroxide, nitric acid, cerium chloride hepta hydrate, stearic acid, ethanol, sodium chloride and calcium hydroxide and universal indicator were procured from Merck, Korea. All formulations were created with analytical-grade chemicals in distilled water.

### 2.2 Development of novel HSC film for steel–cement interface

Before the experiment, the specimens were manually stripped with SiC-500 and SiC-1000 mesh sheets for degreasing. Afterwards they were cleaned in an ultrasonic bath with acetone, isopropyl alcohol (IPA), and deionized (DI) water for 10 minutes each. The preprocessing operation also included alkaline cleaning with Turco 6849 (20 volume percent) at 50 °C. After that, the samples were washed in DI water for 2 minutes.

The conversion HSC film was formed in three steps: (i) oxide growth – the specimen was placed in a 250 mL acidic solution containing H_2_O_2_ (2.5 mL) and HNO_3_ (5 mL) for 30 min to commence the formation of a metal oxide structure on the steel surface. The acidic solution had a pH of 3.5. (ii) Thickening of oxide – the specimens were immersed in DI water for 30 min at 90 °C. The temperature was raised to enhance oxide thickening. (iii) Sealing oxide layer – A 500 mL sealing coating solution was produced by dissolving 10 000 ppm–100,000 ppm CeCl_3_·7H_2_O. The steel pin specimens were immersed in this solution for 60 minutes at 50 °C.

After this, the specimens were placed in another container with a 0.02 M solution of stearic acid in ethanol for 8 minutes at room temperature. Following the coating procedure, the specimens were stored at room temperature for 24 h to dry in ambient air.

**Fig. 2:**
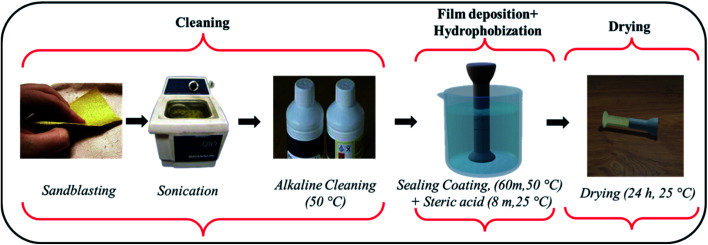
Scheme for deposition of hydrophobic sealing coating film by an immersion process.

All immersive experiments were carried out following the American Society for Testing and Materials (ASTM) G 31-72 and (ASTM) G1 standard criteria. Fig. 2 represents the scheme for deposition of HSC film on steel (pins). The cement block was sprayed (twice) with a coating solution of CeCl_3_·7H_2_O (60 000 ppm), followed by spraying (twice) with a 0.02 M solution of stearic acid in ethanol and cured at room temperature (25 °C).^9,12,16^

### 2.3 Development of concrete pore solution and chloride corrosive media

To evaluate the inhibition characteristics of the HSC film, coated specimens (pin and cement) were dipped in simulated concrete-pore accelerated corrosive medium and chloride medium (0.1–0.6 M NaCl) for 30 days respectively. The simulated corrosive media were prepared from Ca(OH)_2_ (0.03 mol L^−1^) with 0.1–0.6 M NaCl added.^16^ For each set of studies, many samples and corrosive media were investigated to assess the repeatability and reproducibility of the observed results.

### 2.4 Structural characterization of the developed material HSC coating

#### 2.4.1 Scanning electron microscopy (SEM) (determination of surface morphology)

The morphology and structure of the HSC film were determined with an ultra high-resolution scanning electron microscope (S-4800, Hitachi, Japan) equipped with an EDS (FESEM III/EDS, JSM7500F).

#### 2.4.2 Secondary ion spectroscopy (SIMS) (determination and optimization of surface layer modeling)

Time-of-flight secondary ion mass spectrometry (TOF-SIMS) was performed using an ION-TOF SIMS V equipped with a 30 keV Bi primary ion gun and an O_2_ ion sputter gun. Depth profiles were measured in non-interlaced mode. For all the coated specimens the sputter gun was operated at 3 keV.

### 2.5 The functionality of the HSC coating material characterization

#### 2.5.1 Potentiodynamic polarization curves (PDC) (determination of anti-corrosion efficacy)

Based on electrochemical studies, the potentiostat/galvanostat model WIZEIS – 1200 PREMIUM was used. Ag/AgCl, platinum, and pin (specimens) were used as a reference, counter, and operating electrodes, respectively. Tafel extrapolation was used to obtain the polarization parameters. The potential was scanned at 1 mV s^−1^.

#### 2.5.2 X-ray photoelectron spectroscopy (XPS) (determination of chemical configuration and anticorrosion efficacy)

XPS was carried out using a Thermo, ESCALAB250 instrument. The base pressure in the UHV chamber was kept constant at 10^−9^ mbar. To analyze the surface composition of the specimens, spectra were collected at 15 kV and 20 mA using the MgKα (1253.6 eV) radiation line. C 1s was used as a reference for internal binding energy.

#### 2.5.3 Determination of mechanical strength (hardness detection test)

The film's hardness on steel (pin)–cement was determined using a Vickers micro-hardness tester (Shimadzu/HMW-2). The loading force was 90 mN, and the loading time was 10 seconds. For hardness-tested specimens, an OM (Korean Scientific Inc. UCMOS03100KPA) was utilized to view the structure of the indent.

In addition, an X-cut tape test was carried out on the substrates to confirm the interface strength of the film–substrate system. Two slits were produced in the preventive film with fine-edged cutting equipment that were approximately 40 mm long and 0.029 mm deep and crossed near their midpoint, resulting in angles of 30° and 45°. At the intersection of the cuts, a pressure-sensitive clear tape (40 mm long, 12 mm wide) with an adhesive peel strength of 6.34 N cm^−1^ was applied. The surface of the tape was rubbed hard with pressure until the color became homogeneous. The tape was removed after 90 seconds of application by grasping the free end and drawing back at an angle of 180°. The X-cut region was examined to ensure that the sealing coating had been removed from the substrate.

2D modeling of the pin–cement interface was performed using a mechanical damage model for the oxide-jacking resistance of the HSC film.

#### 2.5.4 Contact angle measurements (determination of hydrophobic characteristics)

Contact angle measurements were performed post immersion by using Phoenix 300 Touch with an accuracy of 0.1 degrees. The measurement was performed following ASTM D5946 by using a distilled water drop of 0.003 mL. The computed contact angle readings were over an average of 5 runs.

#### 2.5.5 Universal indicator test (determination of carbonation resistance of HSC flame)

To test the carbonation resistance of the steel (pin)–cement interface, the treated insulator specimen was sealed in an accelerated carbonation chamber for 60 days. The environment was adjusted to a temperature of 20 ± 1 °C, relative humidity of 70 ± 1%, and CO_2_ concentration of 20 ± 1%. Universal indicator was used to evaluate the carbonation resistance of the hydrophobic HSC film.

#### 2.5.6 Determination of the biological-based anticorrosion efficacy

##### 2.5.6.1 Bacterial strains and growth condition applied in the study

*Acidithiobacillus ferroxidans* ATCC 23270 and *Thiobacillus organoparus* Markosian ATCC 27977 strains were used to investigate the antimicrobial potential of the HSC film. Both the bacterial strains were subcultured in brain heart infusion (BHI) broth at 30 °C for 24 h, respectively.

##### 2.5.6.2 Antimicrobial assay

An in-use test and agar well diffusion method were applied to screen the antimicrobial activities of the HSC film. Sodium metabisulfite (Sigma-S1516) (Na_2_S_2_O_2_) was employed as a positive control and DMSO as a negative control. Briefly, 100 μL of fresh bacteria were pipetted into the center of agar plates (Muller–Hinton agar (MHA)). The agar well (8 mm) was aseptically placed on the surface of the agar. The agar well diffusion assay was performed using 100 μL of HSC coating solution (60 000 ppm). The BHA agar plates were incubated at 30 °C for 24 h, and examined for inhibition zones. Antimicrobial activity was determined by measuring the zone of inhibition (including the good diameter). Antimicrobial tests for both bacteria were conducted in duplicate (2 Petri dishes) and the antimicrobial data were recorded as mean values of 2 Petri dishes.

##### 2.5.6.3 Determination of minimum inhibitory concentration (MIC)

The MIC was defined as the lowest concentration of HSC film solution which exhibited clear fluid with no evolution of turbidity.^17^ The MIC of the selected HSC film was quantified according to the method of Bussmann *et al.* using a 48-well microplate. The MIC was performed for *Acidithiobacillus ferroxidans* ATCC 23270 and *Thiobacillus organoparus* Markosian ATCC 27977. Briefly, 0.5 mL of the sterilized BHA broth was filled into the 7 wells of each row. Sequentially, an additional 0.5 mL of a mixture of culture medium (10^6^ CFU mL^−1^) and HSC coating solution was serially added to make a concentration range from 10 000 to 100 000 ppm per 500 mL and well 1 served as the growth control. The microplate was then incubated at 30 °C for 24 h and the resulting turbidity was observed using a spectrophotometer at the optical density (OD 600 nm).

##### 2.5.6.4 Anti-adhesion analysis

To study the effect of growth media and incubation time, a biofilm was grown on 12-well polystyrene microtiter plates (flat bottom) (SPL Life Sciences, Korea) with SS coupons (ASTM A653 G90), surface finish 2B (18 × 18 mm^2^), as described in.^18^ In summary, each well was filled with 200 μL of BHI (MB cell, Korea) and inoculated with 1% (v/v) (10^7^ to 10^8^) culture grown overnight. The plates were then wrapped with parafilm to prevent evaporation during incubation. These plates were incubated at 30 °C for 24 h under static conditions. Biofilm formation was quantified using live (green) Syto 9 (excitation/emission maxima of 485/498 nm) and dead (red) cells propidium iodide (excitation/emission maxima of 493/636 nm) staining with a LIVE/DEAD™ BacLight™ Bacterial Viability kit and the total biomass was estimated, as reported previously.^19^

## 3. Results and discussion

### 3.1 Analysis of steel (pin)–cement interface of aged insulators

**Fig. 3:**
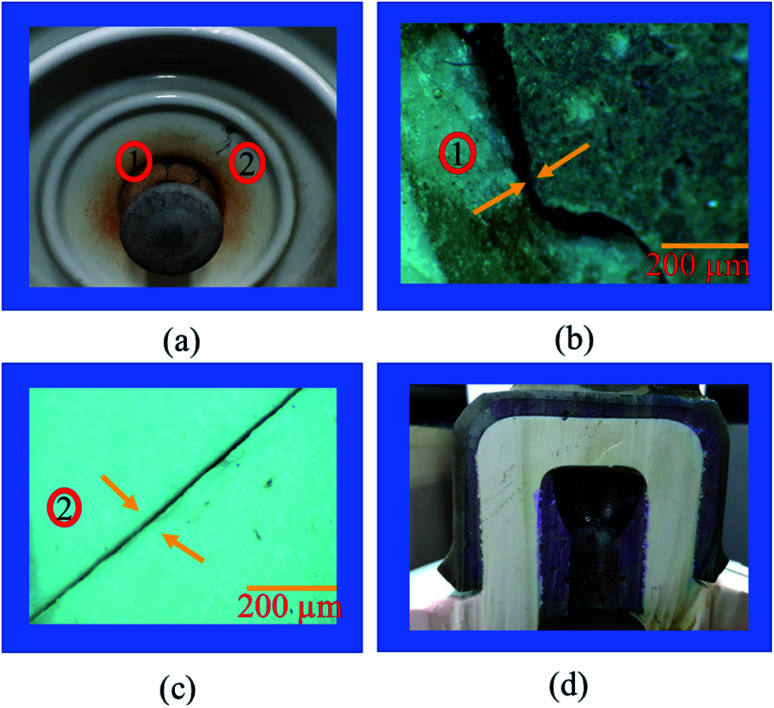
Evaluation of aged insulator specimens: (a) cracks in pin–cement and cement–porcelain interfaces, (b) micrograph of cracked pin–cement interface, (c) micrograph of cracked porcelain, (d) macrograph of carbonated cement of pin–cement interface.

Insulators from various sites in Korea, active for more than 40 years, were collected and evaluated. It is observed that most of the specimens that represented pin corrosion showed cracks in the pin–cement–shell interface and shell (see Fig. 3(a)). The micrographs (see Fig. 3(b)) of the pin–cement–shell interface depicted cracks (105 μm). The reason may be bursting pressure exerted by corrosion byproducts formed on the pin–cement interface.

The micrograph represented in Fig. 3(c) depicts crack propagation from cement to shell. The reason may be the propagation of bursting pressure developed due to corrosion products. The pin–cement interface of the specimens was evaluated to examine the extent of carbonation in the cement (see Fig. 3(d)). The color is closer to the bluish-purple shown for a carbonated specimen (pH: 8). This indicates that the pin–cement interface of the specimen is highly carbonated.

### 3.2 Morphology and composition analysis of HSC film

**Fig. 4:**
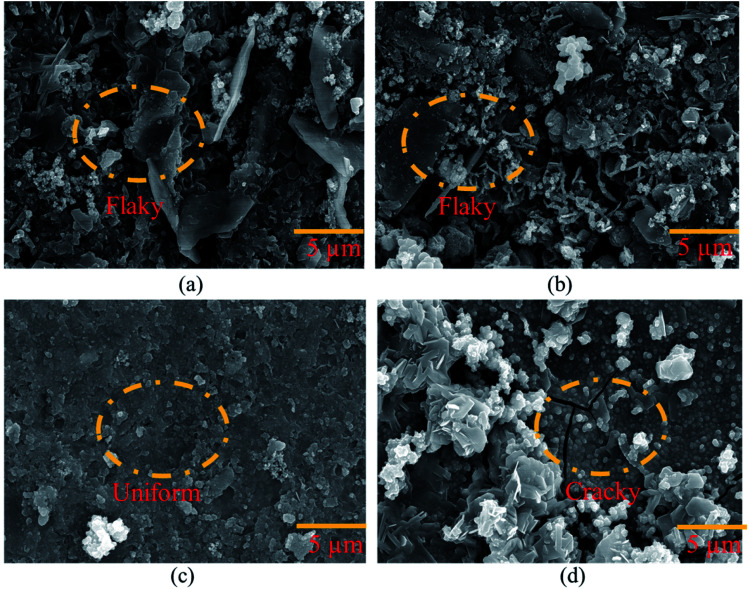
Morphological analysis of the coating developed on the pin of the steel (pin)–cement interface by varying CeCl_3_·7H_2_O: (a) 10 000 ppm, (b) 30 000 ppm, (c) 60 000 ppm, and (d) 100 000 ppm.

After being removed from the coating bath and dried for 24 hours, the HSC film characteristics were examined. Fig. 4 depicts the morphology of coated insulator (steel) pins obtained after immersion in 10 000 ppm to 100 000 ppm CeCl_3_·7H_2_O concentration solutions. Fig. 4(a) and (b) show scanned microscopic micrographs of coated insulator pins after 60 minutes in 10 000 ppm and 30 000 ppm Ce salt baths, respectively. In both situations, a non-uniform and flaky layer developed. This film did not continuously cover the entire surface. The cause might be a low concentration of CeCl_3_·7H_2_O in the sealing coating bath solution. The trials were carried out by gradually increasing the time to 180 minutes of exposure. However, even after extending the exposure period, the data did not indicate any improvement. Fig. 4(c) shows a continuous, crack-free, and homogeneous protective coating produced on the surface of the specimen. The continuous film indicates the filling effect, which may have resulted in the filling of fissures in the cement of the pin–cement contact, interfering with the diffusion of CO_2_ and H_2_O.^27^

Cracks may be seen on the HSC surface in Fig. 4(d). The cause might be strain induced by the creation of a much thicker sealing film.^28^ The fracture progression on the coating surface was caused by the strain that formed within the HSC layer.

**Fig. 5:**
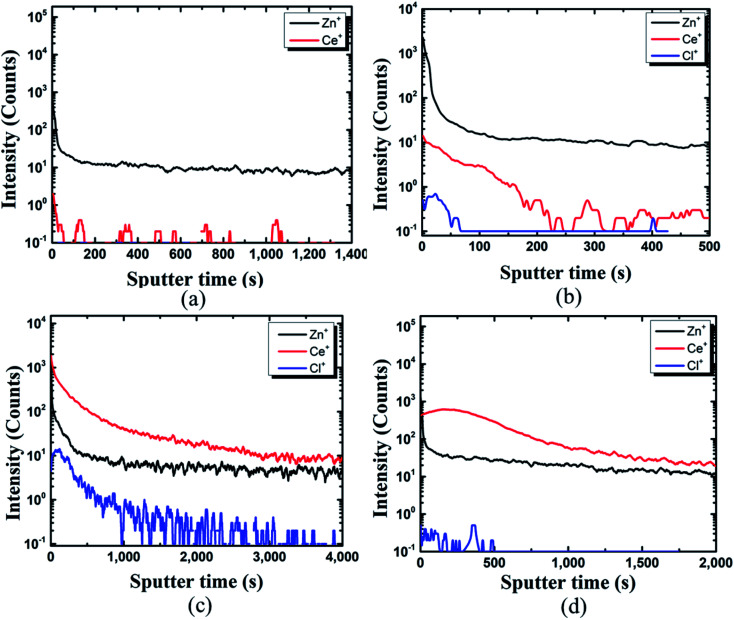
TOF-SIMS analysis of coating developed on the pin of the steel (pin)–cement interface by varying CeCl_3_·7H_2_O: (a) 10 000 ppm, (b) 30 000 ppm, (c) 60 000 ppm, (d) 100 000 ppm.

The TOF-SIMS depth profile of coating film on the pin was studied to gain more insights. The presence of Ce, Zn, and Cl could be identified from the coated pin surface (see Fig. 5(b–d)). In the case of Fig. 5(a), due to the deficiency in Ce ions in the coating bath (10 000 ppm), the protective film was not formed, and thus it could not be detected on the pin surface. In Fig. 5(b), Ce ions could be detected on the pin surface. However, the protective film was not formed on pin specimens, as seen in Fig. 5(b).

The structure could be assigned as Zn^+^/Ce^+^/Cl^+^. With an increase in Ce ions in the coating bath the formation of a protective layer over the Zn surface can be seen from Fig. 5(c) and (d). TOF-SIMS confirms the structure of the coated pin specimens for Fig. 5(c) and (d) as Ce^+^/Zn^+^/Cl^+^. The results of SEM and TOF-SIMS are in agreement with each other.

### 3.3 Inhibition performance of HSC film in the concrete pore solution

**Fig. 6:**
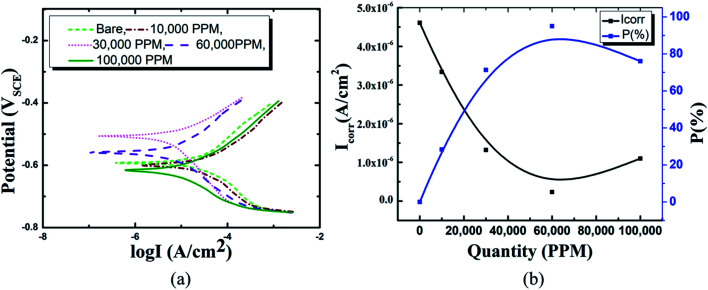
Electrochemical analysis results with various concentrations of cerium ions in concrete media: (a) Tafel curves, (b) variation in *I*_corr_ and anticorrosion performance of HSC film with various concentrations of Ce ions.

Following the synthesis of the HSC film, the inhibitory features of the HSC film were tested using polarization curves. Fig. 6 depicts the electrochemical behavior of HSC film after 30 days of exposure in simulated concrete solution. The Tafel behavior of these specimens is predicted for the anodic and cathodic polarization branches. To determine the optimal content of cerium in the HSC film bath, corrosion kinetic characteristics in the 10 000 ppm–100,000 ppm concentration range were investigated, and inhibition performances, *P* (%), were computed.^29^

In comparison to coated specimens, uncoated specimens have the highest corrosion current density (*I*_corr_), 4.6 × 10^−6^ A cm^−2^. This demonstrates the better corrosion resistance of coated pin specimens (see Fig. 6(a) and (b), ESI Table 1†). With increasing concentrations of Ce salts in the HSC solution, the corrosion current density (*I*_corr_) of the coated specimens falls. *I*_corr_ is lowest (2.3 × 10^−7^ A cm^−2^) and *P* (%) is highest in specimens submerged in a 60 000 ppm Ce-based HSC solution. However, when the concentration of CeCl_3_·7H_2_O hits 100 000 ppm, *I*_corr_ rises (see Fig. 6(a) and (b)). This demonstrates that an excessive concentration of Ce salts in the HSC film may have reduced the fracture resistance and consistency of the protective HSC film. The fissures might have contributed to a rise in *I*_corr_. The corrosion current density (*I*_corr_) for coated specimens is approximately three times lower than for non-coated specimens (see Fig. 6(b)). The inhibition performances (*P* (%)) of HSC films can be calculated using eqn (1).1
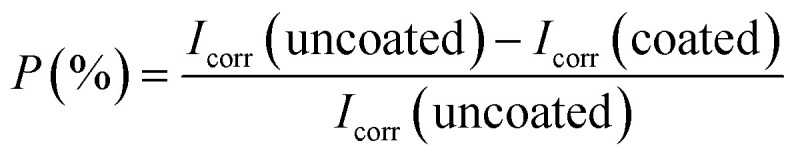


**Fig. 7:**
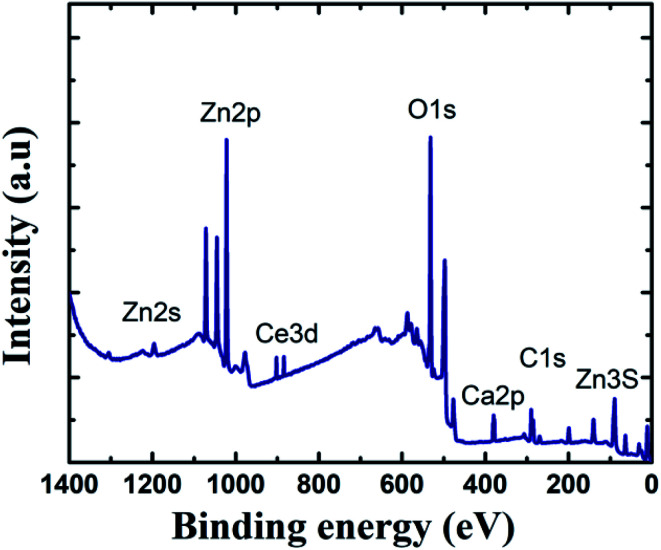
XPS survey spectra of HSC film after 30 days immersion in concrete solution.

To learn more about the inhibitory capabilities of HSC film, XPS tests were performed on specimens after immersion in concrete pore solutions. Fig. 7 depicts the survey spectra for HSC-coated specimens after immersion in a concrete solution. The primary elements found in HSC-coated specimens were Zn, Ce, Na, Ca, and C.

**Fig. 8:**
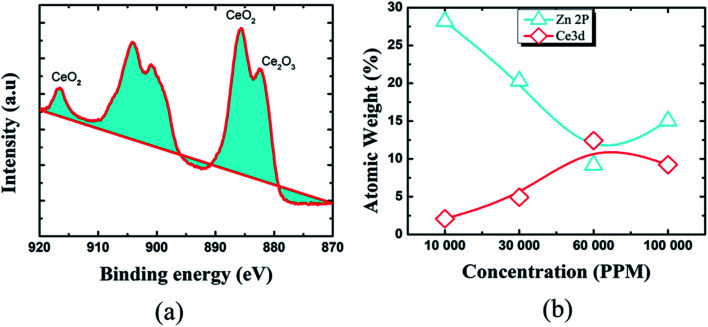
XPS results representing: (a) narrow spectra (Ce 3d), (b) weight (%) of various elements.

The Ca is present due to immersion in concrete solution. The Zn 2p peaks (Fig. 7), with binding energies of 1045.7 eV and 1022.01 eV, represent a ZnO-type complex in specimens. Similarly, the existence of ZnO or a ZnO-type compound in specimens is revealed by the Zn 3s peak at 140.09 eV. At 532.16 eV, O 1s exhibits overlapping peaks showing the presence of both zinc–oxygen and cerium–oxygen. The O 1s signal (532.2 eV) in coated specimens may contribute to cerium-oxide/cerium-hydroxylated compounds. The first signal, correlating to Ce 3d (Fig. 8(a)), with binding energy 881.45 eV, suggests Ce_2_O_3_. The second peak, which matched Ce 3d_5/2_ spectra with a binding energy of 881.70.5 eV, might imply CeO_2_. The existence of CeO_2_ is predicted by the peak at 917 eV. When the theoretical Ce spectra values provided in the literature are compared, it may be concluded that Ce(OH)_3_, CeO_2_, and Ce_2_O_3_ were detected in the Ce-coated specimens.^30–33^

The XPS analyses also reveal the existence of cerium species on all coated pin surfaces after 30 days of immersion in a concrete medium. When the concentration of Ce in the HSC solution grew to 60 000 ppm, the atomic weight (percent) of Ce grew from 2.08 to 12.42, suggesting the production of insoluble cerium compounds on the pin surface (see Fig. 8(b)). A high concentration of Ce in the HSC bath, on the other hand, would result in fissures in the protective film. As a result, the corrosion resistance of the protective coating is reduced.^34–36^

### 3.4 Mechanical performance of the HSC film

**Fig. 9:**
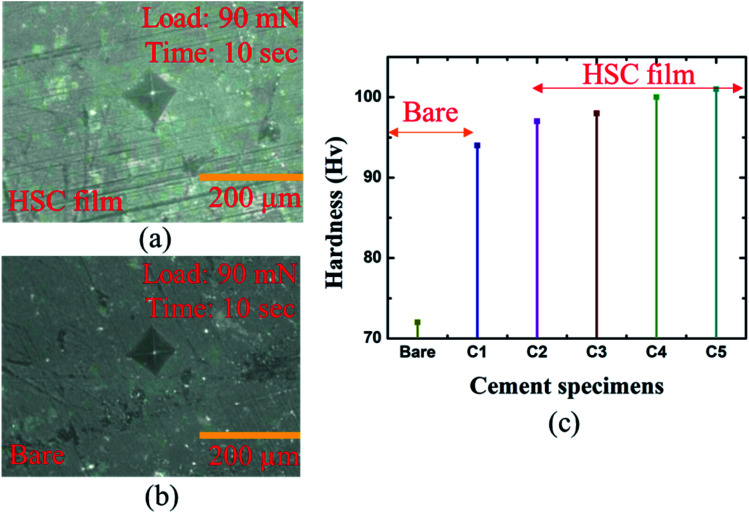
Micro-hardness analysis of: (a) bare cement specimen, (b) HSC-coated specimen, (c) bare and coated cement specimens.

A microhardness test was performed to assess mechanical strength of the coated specimens in hostile chloride settings (see Fig. 9(a–c)). The hardness of the coated specimens was 16.02% greater than that of the bare specimens (see Fig. 9(c)).

The highest hardness of the specimens coated with HSC film was 109 Hv. Specimens C1–5 refer to coated cement specimens. The average hardness of the HSC-coated cement specimens was observed to be 99.6 Hv.

In addition, an X-cut tape test performed confirmed the adhesive strength of the HSC film and substrate was 5A level according to ASTM-D3359 standards, which indicates an excellent combination of substrate and protective film, as represented by ESI Fig. 1 and Table 2.†

Further simulation was performed to analyze the stress and mechanical surface damage propagation of the steel (pin)–cement interface to evaluate the crack resistance of HSC film. In the present article, a 2D model for oxide jacking in the pin–cement interface was performed. The steel (pin)–cement interface was modeled as linear elastic materials, with stress at each time step based on the propagation of corrosion. The effect of stress due to corrosion on the pin was modeled as a scalar damage model. The damage initiates in the steel (pin)–cement interface when the stress crosses the tensile strength.

**Fig. 10:**
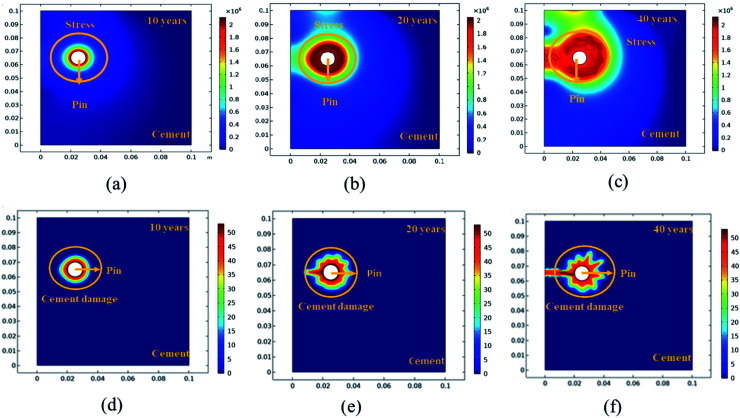
The 2D modeling of: (a–c) stress propagation (N m^−2^) in the steel (pin)–cement interface for uncoated specimens with 10–40 years of service, (d–f) cement surface damage (%) in the steel (pin)–cement interface for uncoated specimens with 10–40 years of service.

**Fig. 11:**
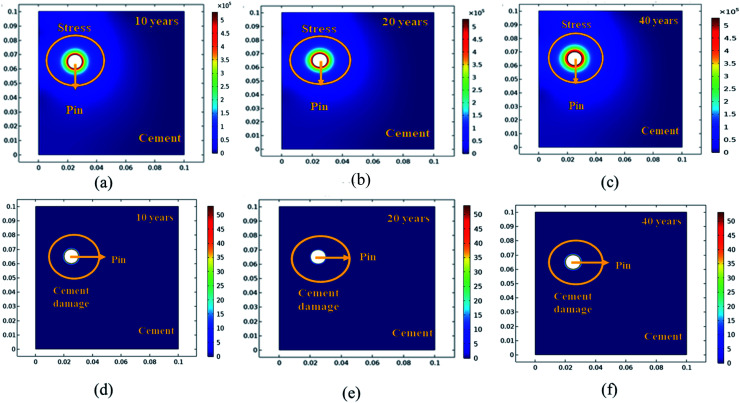
The 2D modelling of: (a–c) stress propagation (N m^−2^) in the steel (pin)–cement interface for HSC coated specimens with 10–40 years of service, (d–f) cement surface damage (%) in the steel (pin)–cement interface for coated specimens with 10–40 years of service.

In addition, a fracture forms when the stiffness of the material deteriorates. The cement surface damage (cracks) was distributed throughout the limited width of the domain. 2D modeling of stress and surface damage propagation in steel (pin)–cement due to deposition of corrosion byproducts after 10–40 years of service is represented in Fig. 10(a–f). The material assignments were specified for the pin–cement interface. The material parameters necessary for the computation of stress fields in each component are Young's modulus and Poisson's ratio. The critical zone with the greatest stress values is seen at the pin–cement contact (see Fig. 10). The maximum stress (red zone), calculated for untreated specimens was 2 × 10^6^ N m^−2^. The stress fields are considerably affected by steel (pin) corrosion and enhanced with increasing (10–40 years) service age of the insulators (see Fig. 10(a–c)). After 40 years of service, the pin–cement interface model represents maximum stress (2 × 10^6^ N m^−2^, see Fig. 10(c)) throughout the pin–cement interface, that propagates to the cement surface. The surface damage was calculated by using the Rankine stress model. The damage model and damage evolution inputs that were considered for surface damage calculation were the scalar damage and linear strain-softening. The calculated cement surface damage (%) for insulators within the service age range 10–40 years increases with increasing service age (see Fig. 10(d–f)). The cause might be an increase in corrosion with service time. After 40 years of service the untreated specimen shows 50% surface damage (see Fig. 10(f)). The stress distribution and cement surface damage modeling of the treated specimens can be seen in Fig. 11(d–f). The maximum stress calculated for treated specimens (see Fig. 11(a–f)) was 5 × 10^5^ N m^−2^. Even after 40 years of service, the specimens do not show stress propagation throughout the cement surface (see Fig. 11(c)). The surface damage models of treated specimens depict 0% cement surface damage even after 40 years of service (see Fig. 11(f)), rendering it oxide-jacking free and crack free. The material assignment specified for treated and untreated specimens for modeling stress distribution and surface damage after a certain service age is shown in Table 1.

**Table 1:** Summary of important parameters for 2D modeling of steel (pin)–cement interface

Type	Material	Young modulus (Pa)	Poisson's ratio	Density (kg m^−3^)	Electrical conductivity (S m^−1^)
Untreated	Pin (ASTM A653 G90)	2.05 × 10^11^	0.28	7850	NA
	Cement	2.50 × 10^10^	0.2	2300	
Treated (HSC)	Pin (ASTM A653 G90)	2.05 × 10^11^	0.28	7850	
	Cement	2.50 × 10^10^	0.2	2300	
	CeCl_3_·7H_2_O	3.50 × 10^10^	0.24	6.75 × 10^−6^	
Electrolyte	Concrete pore solution				12.99

### 3.5 Waterproof and anti-carbonation performance of HSC film

**Fig. 12:**
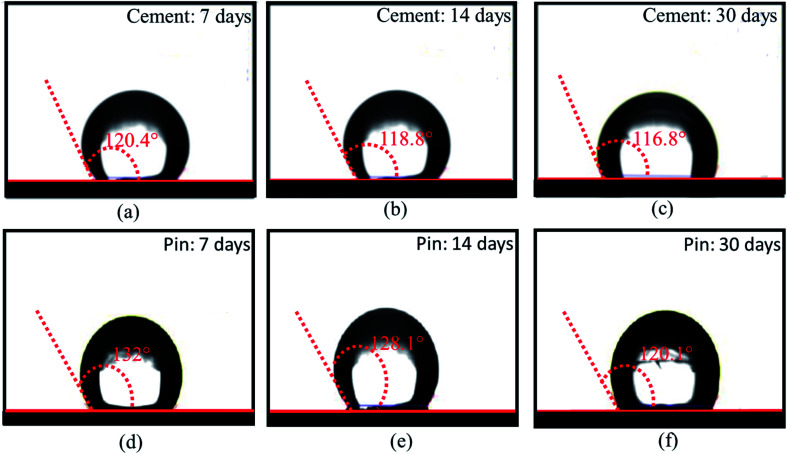
Contact angle measurements of HSC film on cement (a–c) and steel (pin) specimens, (d–f) post-exposure to chloride and concrete pore solution corrosive media for 7,14 and 30 days.

The HSC film improved the waterproof performance of the steel (pin)–cement interface by constructing hydrophobic surfaces. Hydrophobic surfaces involve low surface energy and a distinctive physical shape. The suggested film might considerably improve the micro-roughness of the substrate surface and engulf the air in the cavity, hence increasing the energy barrier that a droplet must overcome while spreading over an uneven surface of the film.^37–39^ As a result, the interface between H_2_O and the pin–cement interface shifts from surface contact to point contact, rendering the HSC film waterproof. To test the waterproof behavior of the HSC film, contact angle measurements were carried after exposure to chloride medium for 30 days. The HSC treatment would slow the passage of H_2_O and Cl^−^ ions inside coatings, extending the coating's protection of the underlying substrate.^40^ After 7–30 days of exposure to chloride solution, the contact angle measurements of the treated cement were found to be 120.4°, 118.8°, and 116.8°, respectively (see Fig. 12(a–c)).

Yet after 30 days of exposure in chloride medium, the drop in contact angle measurements was 2.9% when compared to 7 days of exposure (see Fig. 12(a and c)). The hydrophobic performance of HSC-treated steel (pin) specimens is represented in Fig. 12(d–f). After 7–30 days of immersion in concrete pore solution, the contact angle measurements of treated steel (pin) were found to be 132°, 128.1° and 120.1°, respectively. After 30 days of exposure in concrete pore solution, the treated steel (pin) specimens (see Fig. 12(f)) lost just 9% of their hydrophobicity.

**Fig. 13:**
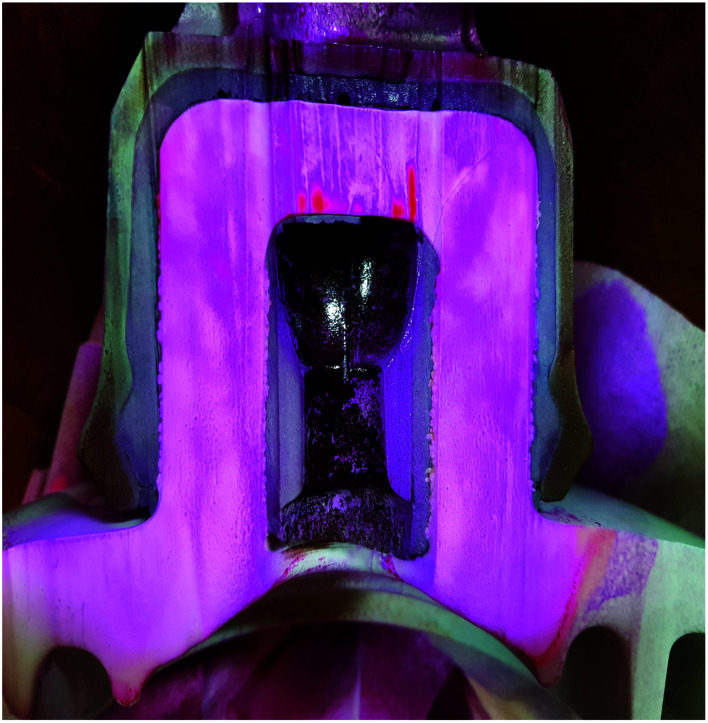
Macrograph indicating the anti-carbonation properties of the HSC film.

After 60 days of exposure to CO_2_ settings, the treated pin–cement interface in Fig. 13 shows a purple hue (pH > 10) due to the use of universal indicator. This demonstrates HSC film's anti-carbonation feature. The HSC film functioned by forming a hydrophobic film on the surface and internal pores of steel (pin)–cement, preventing entry of water-soluble CO_2_.

### 3.6 Antimicrobial activity

Cerium compounds have been renowned as possessing broad-spectrum antimicrobial properties.^41^ In particular, cerium(iii) compounds have been reported to hinder a wide range of pathogenic microorganisms.^42,43^ The mechanism of the CeCl_3_ based antimicrobial action on microorganisms is attributed to the inhibition of the DNA replication process and bacterial cell wall damage or disruption by detected β-d-galactosidase which is present in *E. coli*;^44^ however, accurate mechanisms remain to be determined.^45^ Previously several studies based on the antimicrobial efficacy of cerium compounds were reported. However, the proposed HSC film has been developed with a unique approach and has not been discussed previously for its effectiveness towards *A. ferroxidase* ATCC 23270 or *T. organoparus* Markosian ATCC 27977 in terms of anticorrosion efficacy determination.

**Fig. 14:**
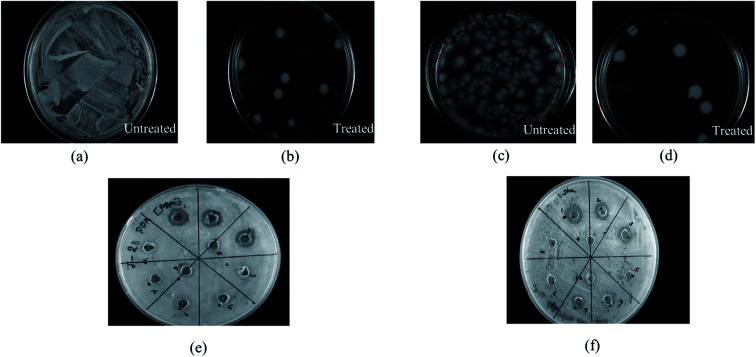
Antimicrobial activity of HSC film evaluated using in-use test and disc diffusion method towards: (a) *A. ferroxidase* ATCC 23270: untreated, (b) *A. ferroxidase* ATCC 23270: treated, (c) *T. organoparus* Markosian ATCC 27977: untreated, (d) *T. organoparus* Markosian ATCC 27977: treated, (e) *A. ferroxidase* ATCC 23270: treated, (f) *T. organoparus* Markosian ATCC 27977: treated.

**Fig. 15:**
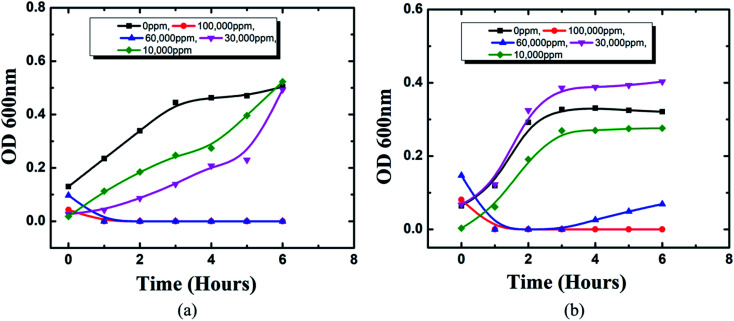
MIC using microdilution assay of HSC film towards: (a) *A. ferroxidans* ATCC 23270, (b) *T. organoparus* Markosian ATCC 27977.

In this study, the antibacterial activities of HSC film were determined using the in-use test and disc diffusion method (Fig. 14(a–f)). In-use studies suggest the lethal effect of HSC film against *A. ferroxidans* and *T. organoparus* Markosian (Fig. 14(a–d)). All HSC coating extracts could hinder the growth of *A. ferroxidans* and *T. organoparus* Markosian; however, HSC film exhibited a more effective hindrance, with the highest zone of inhibition for *A. ferroxidans* ATCC 23270, and *T. organoparus* Markosian ATCC 27977, with diameters of 18.00 ± 0.02 and 21.00 ± 0.08 mm, respectively, as confirmed by the disc diffusion method (Fig. 14(e and f)). Overall, all tested strains were more sensitive to HSC film. The antimicrobial activity exhibited by HSC film was equivalent to that of the positive control sodium metabisulfite; however, no inhibitory activity was observed on the negative control phosphate buffer saline (PBS). Further, the minimum inhibitory concentration (MIC) was determined and evaluated using a microdilution assay and the susceptibility was evaluated using 10 000–100 000 ppm per 500 mL. The antimicrobial effectiveness against *A. ferroxidans* and *T. organoparus* Markosian was highest with 60 000–100 000 ppm per 500 mL HSC coating solution (Fig. 15).

**Fig. 16:**
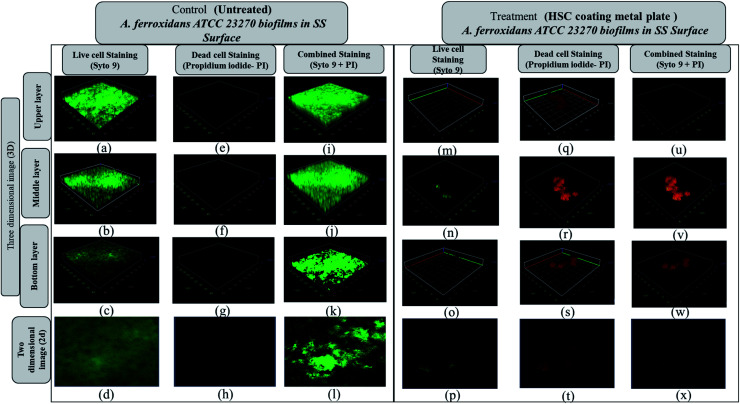
Anti-adhesion analysis confocal scanning microscope 3D and Z-stack images (upper, middle, lower side of each set (3-dimensional image and 2-dimensional image, respectively)) of live (a–d), dead (e–h), and combined live and dead cells (i–l) of *Acidithiobacillus ferroxidans* ATCC 23270 biofilms developed on stainless steel (SS) surfaces in BHI at 25 °C for 24 h. The treated metal plate after incubation showed reduced biofilm (m–x). A bacterial viability kit was used.

The *Acidithiobacillus ferroxidans* ATCC 23270 strain was used to investigate the biofilm detachment potential of HSC-film-coated stainless-steel coupons (ASTM A653 G90, surface finish 2B), 18 × 18 mm^2^. A biofilm was formed on 12-well polystyrene plates at 30 °C. Growth media, incubation time, and the strains used greatly influenced *Acidithiobacillus ferroxidans* ATCC 23270 biofilm formation, as elucidated by a confocal scanning electron microscopy assay (Fig. 16(a–d)). In BHI, after 24 h, *A. ferroxidans* ATCC 23270 strain testing showed biofilm formation ability with live (green) Syto 9 (Fig. 16(a–d)) and dead (red) cells propidium iodide (Fig. 16(e–h)) stained with a LIVE/DEAD™ BacLight™ Bacterial Viability kit. The untreated specimens were compared with the treated ones (Fig. 16(m–x)). The results of the anti-adhesion analysis strongly indicate that the biofilm formation ability of *A. ferroxidans* is greatly affected by the anti-adhesion and antimicrobial efficacy of HSC film. The treated specimens include around 0.1% viable cells (see Fig. 16(m–p)). The anti-adhesion characteristic of HSC film prevents living cells from adhering. Even if living cells attach, the HSC film destroys the maximum number of live cells (see Fig. 16(u–x)).

**Fig. 17:**
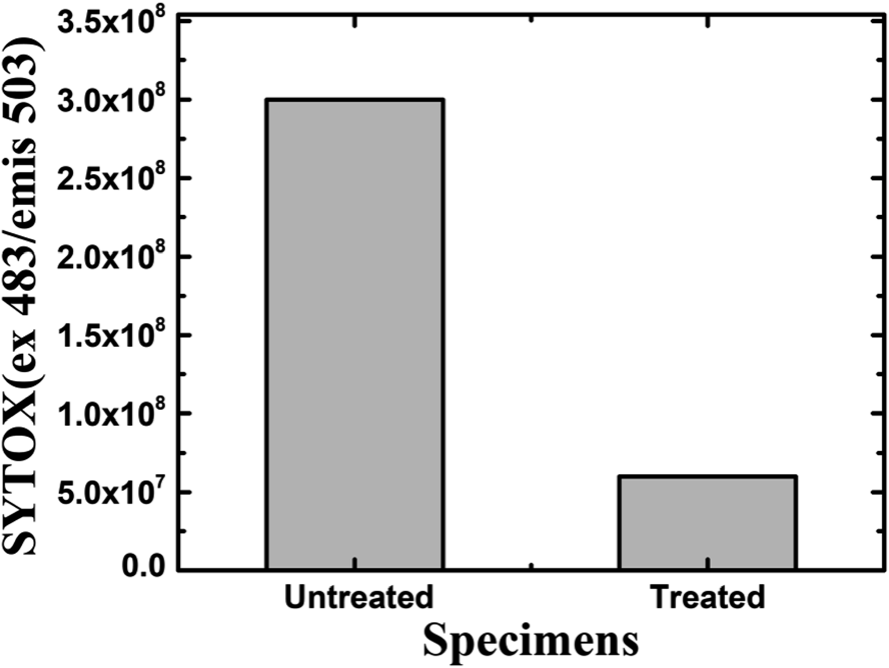
Fluorescence quantification of living cells in the biofilm of *A. ferroxidans* ATCC 23270 strain.

Furthermore, a living cell fluorescence (see Fig. 17) study reveals that treated specimens (HSC film, 60 000 ppm) have an 83.3% lower intensity of living cells.

### 3.7 Proposed mechanism for crack resistance at the steel–cement interface due to HSC film applied at the steel (pin)–cement interface

**Fig. 18:**
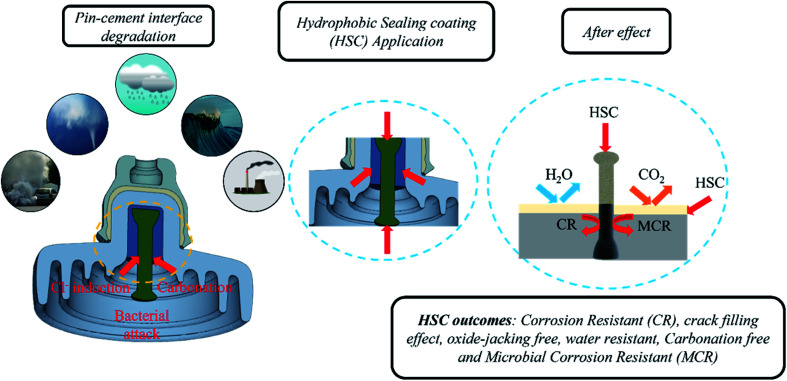
The function of HSC film applied on the steel (pin)–cement interface.

Airborne sea spray, storms, acid rain, and corrosion-inducing bacteria, as well as cement carbonation, are principally responsible for triggering an aggressive corrosion process by destroying the passive coating on steel (pin). Carbonation reduces the alkaline content and pH values of the cement, thus degrading the passive film on the surface of the steel (pin). The HSC film formed a uniform, crack-free, highly dense film on the cement and steel (pin)–cement interface, separating the steel (pin)–cement interface from the external environment, inhibiting the diffusion of CO_2_ and H_2_O into the pores of the steel (pin)–cement interface (see Fig. 18).

In addition, airborne sea spray and storms cause the diffusion of Cl^−^ inside cement pores or bound to the cement hydrate chemically or physically along the path of diffusion.^20–22^ The free Cl^−^ ions may diffuse into the steel (pin) and fracture the passive film, resulting in accelerated corrosion in the steel (pin)–cement interface. The loss of electrons on the steel (pin) surface rises due to corrosion. The presence of H_2_O_2_ in the HSC film enhances reduction or oxidization to OH^−^/O_2_, thereby generating more Ce(OH)_2_^2+^. The Ce^3+^ and Ce^4+^ precipitate to form a thick dense layer of Ce precipitate on the steel (pin), securing the passive film over the steel (pin) from fracture. Thus, it inhibits Cl^−^-induced corrosion in the steel (pin)–cement interface. The precipitation process of Ce^3+^ and Ce^4+^ ions increases the pH of the vicinity (the cement pore solution) in the range 10–12.^23^ Thus, HSC would also inhibit corrosion in the steel (pin)-cement interface of already carbonated cement by interfering in the process of pH drop and thus protecting the steel (pin) against corrosion. The equations supporting the mechanism are as follows.

2 Zn → Zn^2+^ + 2e^−^

3 H_2_O_2_ + 2e^−^ → 2OH^−^

4 2H_2_O_2_ → 2O_2_ + 2H_2_O

5 4Ce^3+^ + O_2_ + 4OH^−^ + 2H_2_O → 4Ce(OH)_2_^2+^

6 Ce(OH)_2_^2+^ + 2OH^−^ → CeO_2_ + 2H_2_O

7 Ce^3+^ + OH^−^ → Ce(OH)_3_

8 2Ce(OH)_3_ → Ce_2_O_3_ + 3H_2_O

Bacteria manifest in three phases. 1. Attachment of bacteria 2. Development of the first pit and nodule 3. Nodule and pit maturation.^24–26^ The HSC film provides an anti-adhesion action against bacteria that cause corrosion. As a consequence, the microorganisms were unable to establish a biofilm by binding to the steel (pin)–cement interface. Even if a microorganism binds to the contact, the HSC film would eradicate it. The functional characteristics described in this section are supported by the results discussed in Section 3.

## 4. Conclusion

In this study, the crack resistance characteristic of HSC film applied on a metal (pin)–interface has been investigated. Unlike those exposed for 60 min in 10 000 ppm and 30 000 ppm sealing coating baths, specimens exposed to a 60 000 ppm Ce salt sealing coating bath formed a consistent and continuous protective covering over the pin surface. The continuous film indicates the filling effect, which may have resulted in the filling of fissures in the cement of the pin–cement contact, interfering with the diffusion of CO_2_ and H_2_O. TOF-SIMS confirms the Ce^+^/Zn^+^/Cl^+^ structure of the optimized HSC film-coated specimen. *I*_corr_ is lowest (2.3 × 10^−7^ A cm^−2^) in specimens submerged in a 60 000 ppm Ce-based coating bath. A larger concentration of Ce in the HSC bath, on the other hand, would create cracks in the protective layer. As a result, the corrosion resistance of the protective layer is lowered. The XPS analyses also reveal the existence of cerium species (HSC film) on all coated specimens even after 30 days of immersion in concrete medium.

The hardness of the coated specimens was 16.02% greater than that of the bare specimens. The highest hardness of the specimens coated with HSC film was 109 Hv. Even after 40 years of operation, the 2D modeling of stress and surface damage for the HSC-treated steel (pin)–cement interface indicates 75% less stress and 100% less surface damage. As a result, the resistance of HSC film against oxide jacking is confirmed for 40 years. After 7–30 days of exposure in chloride media and concrete pore solution, the contact angle measurements of treated cement and steel (pins) were found to be 120.4°, 118.8°, 116.8°, and 132°, 128.1° and 120.1°, respectively, proving the waterproof characteristics of HSC film. After 60 days of exposure to CO_2_ settings, the treated pin–cement interface shows a purple hue (pH > 10) due to the use of universal indicator. The in-use test, disk diffusion method, and microbial adhesion test confirm the bacterial resistance property of HSC film. This demonstrates the anti-carbonation feature of HSC film. As a result of the preceding discussion, it is possible to conclude that the environmentally-friendly HSC film contributes corrosion resistance, fissure filling, hardness, oxide-jacking resistance, waterproofing, anti-carbonation, and anti-bacterial-induced corrosion characteristics, resulting in the crack-free higher durability of steel (pin)–cement structure of insulators. The HSC film may be utilized in any steel–cement interface in construction projects, enabling a new age of environmentally responsible coatings for the construction, engineering, and power industries. Furthermore, it can be beneficial for aged steel–cement interfaces by interfering with the degradation process and improving the remaining lifetime of the steel–cement interface.

## Data availability

The data that support the findings of this study are available on request from the corresponding author.

## Funding

This research was funded by the Korea Electric Power Corporation, R20XO03-08.

## Conflicts of interest

There is no conflict of interest to declare.

## Supplementary Material

RA-012-D2RA00747A-s001
