# Supplementary material for: Crack resistance of a noble green hydrophobic antimicrobial sealing coating film against environmental corrosion applied on the steel–cement interface for power insulators
Source: RSC Adv. 2022 Mar 31;12(16):10126–41. doi: 10.1039/d2ra00747a (PMC8968190; doi:10.1039/d2ra00747a)
Supplement: RA-012-D2RA00747A-s001 [file RA-012-D2RA00747A-s001.pdf]

Electronic Supplementary Material (ESI) for RSC Advances.
This journal is © The Royal Society of Chemistry 2022

Supplementary Materials

Sup table 1. Corrosion kinetic parameters with various concentrations of Cerium ions in concrete media

Quantity (PPM)	E_{corr} (V)	I_{corr} (A/cm^2)	C.R (mm/Y)	P(%)
0	-0.572	4.61E-06	5.41E-02	
10,000	-0.599	3.34E-06	3.92E-02	28.3
30,000	-0.52	1.32E-06	1.55E-02	71.37
60,000	-0.560	2.34E-07	2.80E-03	95
100,000	-0.604	1.10E-06	1.29E-02	76.08

To find the appropriate Cerium content in the HSC film bath, corrosion kinetic features were explored in the 10,000 PPM-100,000 PPM concentration range, Corrosion rate (C.R) and inhibition performances, P(%), were computed as represented in Sup table 1. Uncoated specimens had the highest corrosion current density (I_{corr}) and C.R as compared to coated specimens. This reveals that coated pin specimens have improved corrosion resistance. The corrosion current density (I_{corr}) of coated specimens decreases as the concentration of Ce salts in the HSC solution increases. I_{corr} (2.3x10^{-7}A/cm^2) is lowest in specimens immersed in a 60,000 PPM Ce-based HSC solution. Uncoated specimens exhibit the maximum corrosion current density of 4.6x10^{-6}A/cm^2. when compared to coated specimens. This reveals that coated pin specimens are more corrosion resistant.

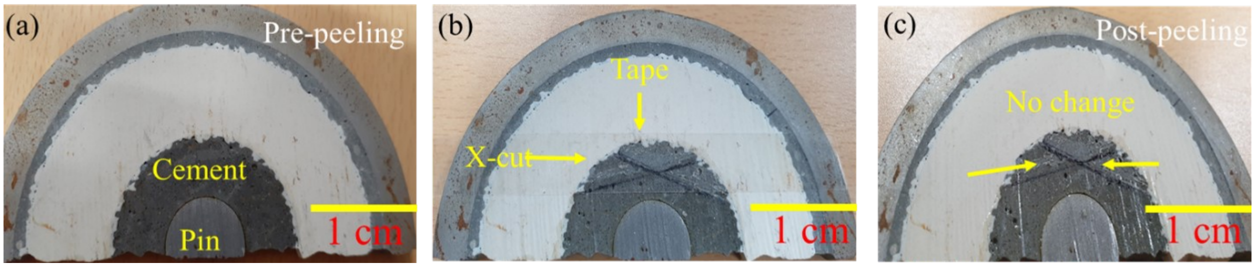

Sup figure 1. Results of the X-cut tape test of HSC (60,000 PPM) treated specimen post immersion in corrosive media for 30 days (a) Before peeling (b) Taped X-cut on specimen (c) Tape peeled from X-cut of specimen

Scale	Indicator
5A	No peeling or removal
4A	Trace peeling or removal
3A	rugged removal along incision (1.6 mm)
2A	rugged removal along incision (3.2 mm)
1A	Removal from most of the area X under tape
0A	Removal beyond the area of the X

Sup table 2. Adhesion rating scale

The X-cut tape test were performed to evaluate the adhesive bonding and interface strength of the coating-substrate system to prevent premature interface failure. The X-cut tape test of the specimen is shown in Figure Sup figure 1. The adhesive strength of the HSC film and substrate was 5A level according to ASTM-D3359 standards, which indicates an excellent combination of substrate and protective film. Adhesion is rated according to the ASTM standards and the scale varies from 0A (Removal beyond the area of incision) to 5A (No peeling or removal). Sup table 2 shows the adhesion rating scale for substrates. Sup figure 1(c) confirms that specimen does not show peeling of the HSC film and thereby passes the adhesive test with the 5A scale.
